# Government Direct-to-Consumer Education to Reduce Prescription Opioid Use: A Cluster Randomized Clinical Trial

**DOI:** 10.1001/jamanetworkopen.2024.13698

**Published:** 2024-05-29

**Authors:** Justin P. Turner, Alex S. Halme, Patricia Caetano, Aili Langford, Cara Tannenbaum

**Affiliations:** 1 Centre for Medicine Use and Safety, Monash University, Parkville, Victoria, Australia; 2 Faculté de Pharmacie, Université de Montréal, Montréal, Québec, Canada; 3 Département de Médecine Spécialisée, Centre Intégré de Santé et de Services Sociaux de la Gaspésie, Sainte-Anne-des-Monts, Québec, Canada; 4 Drug Data Services and Analytics, Canadian Agency for Drugs and Technologies in Health, Ontario, Canada; 5 Faculté de Médecine, Université de Montréal, Montréal, Québec, Canada; 6 Centre de Recherche de l’Institut Universitaire de Gériatrie de Montréal, Montréal, Québec, Canada

## Abstract

**Importance:**

Direct-to-consumer education reduces chronic sedative use. The effectiveness of this approach for prescription opioids among patients with chronic noncancer pain remains untested.

**Objectives:**

To evaluate the effectiveness of a government-led educational information brochure mailed to community-dwelling, long-term opioid consumers to reduce prescription opioid use compared with usual care.

**Design, Setting, and Participants:**

This cluster randomized clinical trial was conducted from July 2018 to January 2019 in Manitoba, Canada. All adults with long-term opioid prescriptions were enrolled (n = 4225). Participants were identified via the Manitoba Drug Program Information Network. Individuals receiving palliative care or with a diagnosis of cancer or dementia were excluded. Data were analyzed from July 2019 to March 2020.

**Intervention:**

Participants were clustered according to their primary care clinic and randomized to the intervention (a codesigned direct-to-consumer educational brochure sent by mail) or usual care (comparator group).

**Main Outcomes and Measures:**

The main outcome was discontinuation of opioid prescriptions at the participant level after 6 months, ascertained by pharmacy drug claims. Secondary outcomes included dose reduction (in morphine milligram equivalents [MME]) and/or therapeutic switch. Reduction in opioid use was assessed using generalized estimating equations to account for clustering, with prespecified subgroup analyses by age and sex. Analysis was intention to treat.

**Results:**

Of 4206 participants, 2409 (57.3%) were male; mean (SD) age was 60.0 (14.4) years. Mean (SD) baseline opioid use was comparable between groups (intervention, 157.7 [179.7] MME/d; control, 153.4 [181.8] MME/d). After 6 months, 235 of 2136 participants (11.0%) in 127 clusters in the intervention group no longer filled opioid prescriptions compared with 228 of 2070 (11.0%) in 124 clusters in the comparator group (difference, 0.0%; 95% CI, −1.9% to 1.9%). More participants in the intervention group than in the control group reduced their dose (1410 [66.0%] vs 1307 [63.1%]; difference, 2.8% [95% CI, 0.0%-5.7%]). Receipt of the brochure led to greater dose reductions for participants who were male (difference, 3.9%; 95% CI, 0.1%-7.7%), aged 18 to 64 years (difference, 3.7%; 95% CI, 0.2%-7.2%), or living in urban areas (difference, 5.9%; 95% CI, 1.9%-9.9%) compared with usual care.

**Conclusions and Relevance:**

In this cluster randomized clinical trial, no significant difference in the prevalence of opioid cessation was observed after 6 months between the intervention and usual care groups; however, the intervention resulted in more adults reducing their opioid dose compared with usual care.

**Trial Registration:**

ClinicalTrials.gov Identifier: NCT03400384

## Key Points

**Question:**

Can a government-led, mailed, direct-to-consumer educational brochure reduce use of long-term prescription opioids among community-dwelling adults?

**Findings:**

In this cluster randomized clinical trial including 4206 adults, there was no difference in the number of people filling opioid prescriptions within 6 months between those who received the educational brochure and those receiving usual care.

**Meaning:**

A direct-to-consumer educational brochure led to no difference in prevalence of opioid cessation compared with usual care among community-dwelling adults taking opioids long term.

## Teaser

This cluster randomized clinical trial investigates whether a government-led, mailed, direct-to-consumer educational brochure reduces prescription opioid use among community-dwelling adults with long-term opioid prescriptions.

## Introduction

Canada and the US have had a substantial rise in opioid-related hospitalizations and deaths over the past 8 years due to illicit and prescription opioid consumption.^1,2,3,4^ Estimates suggest that accidental opioid overdose causes 1 death every 2 hours in Canada.^3^ In 2015, the Canadian federal government proclaimed an opioid crisis, with federal and provincial governments searching for safe and effective ways to reduce prescription opioid use alongside harm reduction strategies to minimize the negative consequences of illicit opioid consumption.^5^

Governments around the world have experimented with policies to diminish opioid prescriptions, implementing a range of clinician-directed interventions to reduce opioid prescribing. Policies that have been attempted include the introduction of opioid use disorder–deterrent formulations, such as extended-release oxycodone,^6,7^ prescription drug monitoring programs,^8,9^ programs to prevent clinician shopping,^10,11^ and prescriber audit and feedback.^11,12,13^ Some policies proved ineffectual or minimally beneficial,^14,15,16^ while others were associated with unintended harms, abrupt opioid cessation, and increased rates of illicit opioid use, suicide, and mental health crises.^17,18,19^ Knowing that many patients with chronic pain will experience improvement in quality of life and function following opioid tapering^20^ but stymied by the dearth of high-certainty evidence to support specific policy interventions, decision makers may grapple with how to proceed.

In the absence of clarity on which policies can safely and effectively reduce long-term opioid prescriptions,^15^ the government of Manitoba examined successful deprescribing trials. Direct-to-consumer education produced a 27% reduction in the use of sedative hypnotics among community-dwelling older adults,^21^ thus raising the question of whether the intervention could be transferable to opioids. Partnering with the Canadian Medication Appropriateness and Deprescribing Network,^22^ a collaborative policy protocol was designed to enable scientific evaluation.^13,22^ The Trial Applying Policy to Eliminate or Reduce Inappropriate Narcotics in the General Population (TAPERING)^23^ was a joint initiative between the provincial drug programs branch at the Manitoba Health, Seniors and Active Living (MHSAL) department and researchers at the Université de Montreal, Québec, to determine the effect of scaling and implementing a direct-to-consumer educational intervention on the cessation and reduction of long-term opioid prescriptions among community-dwelling adults.

## Methods

### Trial Design and Oversight

TAPERING was a prospective, cluster-randomized parallel clinical trial conducted from July 2018 to January 2019 in everyday clinical settings in Manitoba, Canada. The trial protocol has been published previously,^23^ and the full trial protocol and statistical analysis plan are available in Supplement 1. This study followed the Consolidated Standards of Reporting Trials (CONSORT) reporting guideline.^24^ Opioid consumers receiving prescriptions from the same primary care clinic (cluster) were randomized to intervention or control to avoid cross-contamination at the clinic or physician level. The trial underwent 4 approval processes^23^: (1) from the research ethics board of the Centre de Recherche de l’Institut Universitaire de Gériatrie de Montréal, part of the Centre Intégré Universitaire en Santé et Services Sociaux du Centre-Sud de l’Île de Montréal, Canada; (2) from the executive director of the provincial drug programs branch at MHSAL; (3) from the health information privacy committee of MHSAL; and (4) from the Manitoba Monitored Drug Review Committee. Patient data were collected according to provincial privacy legislation and approved by a provincial information access committee; thus, participant informed consent was not required and a waiver of consent was granted by the Health Information Privacy Committee.

### Participants

Eligibility was determined using the Drug Program Information Network (DPIN) prescription claims database at MHSAL, which collects data on all medications dispensed at community pharmacies for the 1.3 million residents of Manitoba regardless of the mode of payment, including date of dispensing, quantity of opioids prescribed, and prescription duration. All residents were screened for prescription opioid use in the 120 days prior to July 1, 2018. Adults aged 18 years or older receiving 90 or more days of opioids (long-term opioid consumers) dispensed from community pharmacies were included. All dispensed opioids excluding codeine and tramadol were eligible for inclusion per the study protocol.^23^ Exclusion criteria included receiving palliative care, receiving treatment for cancer within the previous 12 months, receiving opioids entirely from hospitals, residing in a nursing home, or having a diagnosis of dementia.

### Randomization

On July 1, 2018, prescription data were extracted from the DPIN to identify eligible individuals. Participants were assigned to their primary physician, defined as the physician who wrote most of their prescriptions in the previous 6 months. When possible, physicians with at least 1 eligible patient were clustered within family medicine clinics based on their registered practicing postal code. Cluster units were stratified by number of prescribers (≤10, 11-20, 21-30, 31-40, or >40 per stratum). Physicians with no registered postal code were clustered into 1 stratum.

Clusters were randomized in a 1:1 ratio for each stratum by software-generated pseudorandom numbers. Participants linked to each cluster allocated to the intervention group were dispatched an educational brochure by national mail service in the week following July 18, 2018. Most participants were assumed to have received the document by July 30. The control waiting list group received usual care until brochures were dispatched to them after February 1, 2019.

Blinding of participants was not possible because the educational intervention encouraged participants to discuss their opioid use with their physician. The College of Physicians & Surgeons of Manitoba was asked to alert its members to a public awareness campaign relating to opioids. To maintain privacy and confidentiality, MHSAL conducted the identification of recipients, determination of eligibility, randomization, allocation, and dispatch of the brochures.

### Intervention

A 20-page educational brochure on opioids for chronic noncancer pain was mailed directly to participants along with an individualized cover letter providing context. The brochure was modeled on successful interventions and based on social constructivism and self-efficacy theories.^21,25^ The codesigned brochure was written in both of Canada’s official languages, French and English, and was refined following review by health care professionals and multiple focus groups including community-dwelling adults with chronic noncancer pain. The brochure was sent to all participants in English (the official language of Manitoba); however, French-language versions were available on request, as detailed in the cover letter. Educational material on chronic pain, long-term opioid use risks, self-management strategies, and resources available for tapering were included. The brochure was culturally sensitive for Manitoba’s Indigenous population, included sex- and gender-based content, and was written at an eighth-grade reading level. Readers were instructed to contact their health care practitioner before making a medication change. The brochure and an opioid tapering calculator are available online.^26^

### Data Collection

MHSAL collected baseline demographic characteristics (age, sex, and postal code of primary physician to enable clustering and determine geographic location, defined as urban or nonurban), mortality at 6 months, and DPIN data on all prescription medications, including strength, dose, quantity, date of dispensing, and morphine milligram equivalents (MME) (eTable in Supplement 2). Additionally, MHSAL collected data on nondelivered mail and provided a telephone number so participants could access additional information or provide feedback.

### Outcome Measures

The primary outcome was complete opioid cessation after 6 months, assessed at the individual level and defined as having no opioid prescription dispensed within 60 days^7,8^ and no previous supply with a duration that overlapped this period. Secondary outcomes included dose reduction and/or a therapeutic switch. Opioid reduction was calculated using the MME for all eligible dispensed opioids. Reduction considered the mean daily MME for the month immediately prior to the intervention compared with the mean daily MME for the month immediately following the 6-month follow-up period. Opioid reduction was calculated in 3 ways: (1) any dose reduction, (2) a dose reduction of 25% or greater, or (3) the proportion of participants prescribed at least 90 MME per day at baseline whose prescription was reduced to less than 90 MME per day, based on Canadian guidelines.^27^ Therapeutic switches were identified if an incident prescription for an alternate medication was filled during the 6-month follow-up that had not previously been prescribed during the 12 months prior to the intervention. Alternative medications included other opioids, gabapentin, pregabalin, tricyclic antidepressants, duloxetine, neuropathic agents, disease-modifying antirheumatic drugs, nonsteroidal anti-inflammatory drugs, and biologics. Prespecified subgroup analysis considered the impact of participants’ sex and age (18-64 or ≥65 years).^23^ Nonprespecified post hoc analyses considered the impact of urban vs nonurban location and the effect of the intervention on all-cause mortality.

### Statistical Analysis

A minimum sample size of 940 participants per arm provided 90% power to detect a 5% between-group difference in the primary outcome assuming a 5% opioid discontinuation rate in the control arm, a minimum 10% discontinuation rate in the intervention arm, a 2-sided α set at 0.05, a conservative intracluster correlation coefficient (ICC) of 0.2, and a mean of 5 participants per cluster. All medications were coded by their International Nonproprietary Name and Anatomical Therapeutic Chemical codes as recommended by the World Health Organization.^28^

All outcomes were analyzed in an intent-to-treat fashion. Statistical inference on binary outcomes was based on absolute risk difference between groups after 6 months. For continuous data, between-group differences in preintervention vs postintervention change were computed. For both types of outcomes, the parameter of interest was derived from generalized estimating equations (GEEs) with the appropriate family: binomial or gaussian. This method accounts for the clustering effects. Identity links with exchangeable correlation structures were specified. Each participant was the unit of analysis, while primary care clinics were the cluster. Packages gee, geepack, ICC, and ICCbin in R, version 3.5.3 (R Project for Statistical Computing), were used for GEE and ICC computation, and 95% CIs around parameter estimates were used as the threshold to determine statistical significance. All eligible participants received the educational brochure; however, participants with mean opioid use of more than 2000 MME per day were considered outliers and excluded from the analysis. Data were analyzed from July 2019 to March 2020.

Assessment of opioid-associated mortality was not prespecified because this outcome is determined by the coroner records, which are not linked to MHSAL pharmacy data.^3^ However, all-cause mortality data became available based on the censoring data, enabling a post hoc mortality analysis using the Kaplan-Meier method log-rank test and Cox proportional hazards regression to examine the effect of the intervention on all-cause mortality at 6 months.

## Results

**Figure 1.**
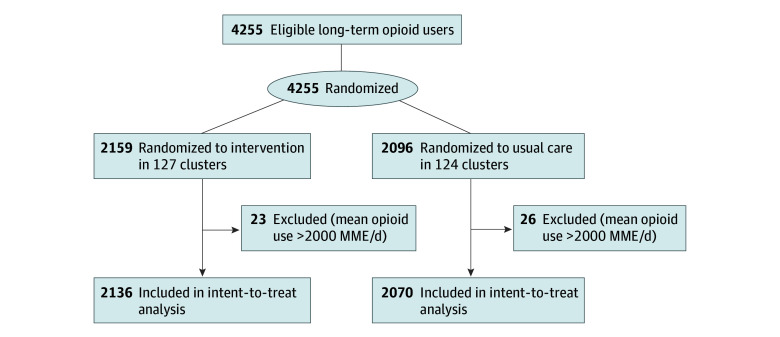
CONSORT Study Flow Diagram MME indicates morphine milligram equivalents.

A total of 4255 adults were eligible for inclusion. After exclusion of 49 participants with mean opioid use of more than 2000 MME per day, the final study population included 4206 individuals; 1797 (42.7%) were female, and 2409 (57.3%) were male. Mean (SD) age was 60.0 (14.4) years (range, 19-100 years) (Table 1). No significant difference in mean (SD) baseline use of prescription opioids or MME was observed between groups (intervention, 157.7 [179.7] MME/d; control, 153.4 [181.8] MME/d) (Table 1). Participants were cluster randomized to the intervention (2136 participants in 127 clusters) or usual care (2070 participants in 124 clusters) (Figure 1). Of 2136 brochures mailed to participants randomized to the intervention, only 40 (1.9%) were returned as undeliverable.

**Table 1.** Baseline Demographics of Participants

Characteristic	Participants^a^		
	Total (N = 4206)	Intervention (n = 2136)	Usual care (n = 2070)
Age			
Mean (SD) [range], y	60 (14.4) [19-100]	60.2 (14.3) [19-100]	59.9 (14.5) [19-99]
>65 y	1337 (31.8)	689 (32.3)	648 (31.3)
Sex			
Female	1797 (42.7)	910 (42.6)	887 (42.9)
Male	2409 (57.3)	1226 (57.4)	1183 (57.1)
Urban	2166 (51.5)	1142 (53.5)	1024 (49.5)
Opioid use			
Any	4206 (100)	2136 (100)	2070 (100)
Fentanyl	617 (14.7)	323 (15.1)	294 (14.2)
Hydromorphone	1894 (45.0)	951 (44.5)	943 (45.6)
Meperidine	47 (1.1)	22 (1.0)	25 (1.2)
Morphine	1052 (25.0)	548 (25.7)	504 (24.3)
Oxycodone	986 (23.4)	487 (22.8)	499 (24.1)
Multiple	376 (8.9)	188 (8.8)	188 (9.1)
Morphine milligram equivalents, mean (SD)^b^	155.7 (179.7)	157.7 (179.1)	153.4 (180.8)

^a^ Data are presented as number (percentage) of participants unless otherwise indicated.

^b^ Calculated as the mean daily morphine milligram equivalents for the previous month, taking into account all opioids dispensed to the participant during the claim period.

For all opioids, the 6-month discontinuation rate in the intervention group was 11.0% (235 participants) compared with 11.0% (228 participants) in the control group (difference, 0.0%; 95% CI, −1.9% to 1.9%) (Table 2). Neither age (18-64 or ≥65 years), sex, nor location (urban or nonurban) significantly impacted opioid cessation.

**Table 2.** Proportion of Participants With Opioid Cessation or Dose Reduction After 6 Months

Opioid reduction outcome	Participants, No./total No. (%)		Absolute difference (95% CI), %
	Intervention (n = 2136)	Usual care (n = 2070)	
**Complete cessation**			
All participants	235 (11.0)	228 (11.0)	0.0 (−1.9 to 1.9)
Age, y			
18-64	152/1447 (10.5)	144/1422 (10.1)	0.4 (−1.8 to 2.6)
≥65	83/689 (12.1)	84/648 (13.0)	−0.9 (−4.4 to 2.7)
Sex			
Female	86/910 (9.5)	93/887 (10.5)	−0.1 (−3.8 to 1.7)
Male	149/1226 (12.2)	135/1183 (11.4)	0.78 (−1.8 to 3.3)
Urban	120/1142 (10.5)	96/1024 (9.4)	1.1 (−1.4 to 3.6)
Nonurban	115/994 (11.6)	132/1046 (12.6)	−1.0 (−3.9 to 1.8)
**≥25% Dose reduction**			
All participants	628/2136 (29.4)	573/2070 (27.7)	1.7 (−1.0 to 4.4)
Age, y			
18-64	420/1447 (29.0)	367/1422 (25.8)	3.1 (−0.1 to 6.4)
≥65	208/689 (30.2)	206/648 (31.8)	−1.6 (−6.6 to 3.4)
Sex			
Female	251/910 (27.6)	240/887 (27.1)	0.5 (−3.6 to 4.6)
Male	377/1226 (30.8)	333/1183 (28.2)	2.6 (−1.1 to 6.2)
Urban	331/1142 (29.0)	256/1024 (25.0)	4.0 (0.3 to 7.7)
Nonurban	297/994 (30.0)	317/1046 (30.3)	−0.5 (−4.5 to 3.5)
**Any dose reduction**			
All participants	1410 (66.0)	1307 (63.1)	2.8 (0.0 to 5.7)
Age, y			
18-64	951/1447 (65.7)	882/1422 (62.0)	3.7 (0.2 to 7.2)
≥65	459/689 (66.6)	425/648 (65.6)	1.1 (−4.0 to 6.1)
Sex			
Female	588/910 (64.6)	559/887 (63.0)	1.6 (−2.8 to 6.1)
Male	822/1226 (67.1)	748/1183 (63.3)	3.9 (0.1 to 7.7)
Urban	770/1142 (67.4)	630/1024 (61.5)	5.9 (1.9 to 9.9)
Nonurban	640/994 (64.4)	677/1046 (64.7)	−0.3 (−4.5 to 3.8)
**Dose reduction to <90 MME/d**			
All participants	194/1115 (17.4)	190/1029 (18.5)	−1.1 (−4.3 to 2.2)
Age, y			
18-64	135/828 (16.3)	134/787 (17.0)	−0.7 (−4.3 to 2.9)
≥65	59/289 (20.4)	56/240 (23.3)	−2.9 (−10.0 to 4.2)
Sex			
Female	81/533 (15.2)	77/492 (15.7)	−0.5 (−5.0 to 3.9)
Male	113/583 (19.4)	113/536 (21.1)	−1.7 (−6.4 to 3.0)
Urban	99/605 (16.4)	87/539 (16.1)	0.4 (−3.9 to 4.6)
Nonurban	95/514 (18.5)	103/485 (21.2)	−2.8 (−7.8 to 2.2)

Abbreviation: MME, morphine milligram equivalents.

More people achieved a dose reduction in the intervention group compared with the usual care group (1410 [66.0%] vs 1307 [63.1%]; difference, 2.8%; 95% CI, 0.0%-5.7%) (Table 2). Significant differences were observed in subgroup analysis: intervention participants who were male (difference, 3.9%; 95% CI, 0.1%-7.7%), aged 18 to 64 years (difference, 3.7%; 95% CI, 0.2%-7.2%), or living in urban areas (difference, 5.9%; 95% CI, 1.9%-9.9%) were more likely to reduce their dose compared with the usual care group (Table 2). Although more people in the intervention group achieved a dose reduction, the between-group difference in mean MME at 6 months was not significant (−19.9 MME/d vs −18.3 MME/d; difference, −1.6 MME/d; 95% CI, −7.3 to 4.1 MME/d).

A similar proportion of participants in both groups achieved a 25% or greater dose reduction (intervention, 628 [29.4%]; control, 573 [27.7%]; difference, 1.7%; 95% CI, −1.0% to 4.4%) (Table 2). In urban areas, 4.0% (95% CI, 0.3%-7.7%) more participants from the intervention group reduced their dose by 25% or more compared with the control group.

Dose reduction to less than 90 MME/d was achieved by 194 participants (17.4%) in the intervention group and 190 (18.5%) in the usual care group who originally consumed 90 MME/d or more, with no significant between-group difference (Table 2). Therapeutic switches occurred at similar rates between groups (Table 3). The most frequent newly initiated medications were other opioids, acetaminophen, tricyclic antidepressants, and disease-modifying antirheumatic drugs.

**Table 3.** Proportion of Participants Who Were Dispensed a New Analgesic During the 6-Month Follow-Up

New medication^a^	Participants, No./total No. (%)^b^		Absolute difference (95%CI), %
	Intervention (n = 2147)	Usual care (n = 2078)	
Acetaminophen	46/445 (10.4)	56/416 (13.5)	−3.2 (−7.6 to 1.1)
Biologic	2/2123 (0.0)	1/2016 (0.0)	0.0 (0.0 to 0.0)
DMARD	80/1477 (5.4)	93/1460 (6.4)	1.0 (−2.8 to 0.7)
Duloxetine	8/1975 (0.4)	5/1876 (0.3)	0.1 (−0.2 to 0.5)
Gabapentin	24/1682 (1.4)	30/1599 (1.9)	−0.4 (−1.3 to 0.4)
Neuropathic agent	39/1954 (2.0)	47/1854 (2.5)	−0.5 (−1.5 to 0.4)
NSAID	2/2082 (0.1)	1/2016 (<0.1)	0.0 (−0.1 to 0.2)
Addition of a new opiate	69/1269 (5.5)	62/1147 (5.4)	0.1 (−0.11 to 0.19)
Pregabalin	30/1879 (1.6)	34/1847 (1.9)	−0.2 (−1.1 to 0.6)
Tapentadol	33/1995 (1.7)	35/1932 (1.8)	−0.2 (−1.0 to 0.7)
Tramadol	22/2017 (1.1)	24/1962 (1.2)	−0.1 (−0.8 to 0.5)
Tricyclic antidepressant	48/878 (5.5)	52/893 (5.8)	−0.3 (−2.5 to 1.8)

Abbreviations: DMARD, disease modifying antirheumatic drug; NSAID, nonsteroidal anti-inflammatory drug.

^a^ Defined as a pharmacy claim for any of the listed medications that had not been claimed in the previous 12 months.

^b^ Percentages are calculated as the number of participants who had a claim for a new medication class during the 6-month follow-up divided by the number of participants who did not have a claim for the medication class in the previous 12 months.

**Figure 2.**
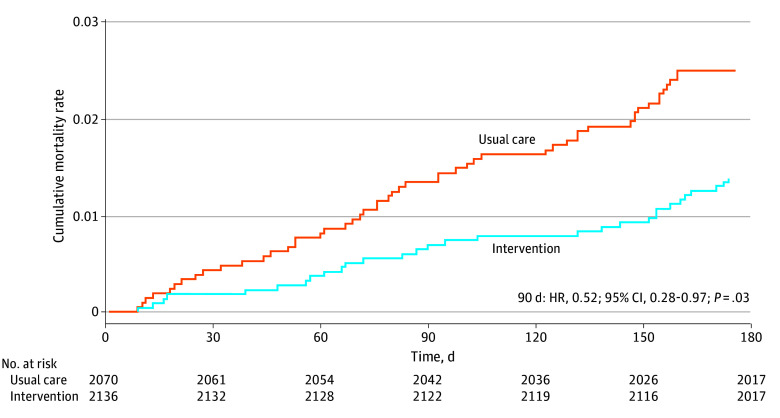
Kaplan-Meier Curves for All-Cause Mortality

More control group participants died (53 [2.5%]) during 6-month follow-up compared with intervention group participants (30 [1.4%]). A significant reduction in all-cause mortality in the intervention group was apparent 90 days after dispatch of the brochures (hazard ratio, 0.52; 95% CI, 0.28-0.97) (Figure 2).

## Discussion

A government-led direct-to-consumer educational intervention produced a modest reduction in the mean daily prescription opioid dose in long-term opioid consumers. However, it failed to show benefit for the primary end point of cessation of opioid prescriptions compared with usual care. Subgroup analyses identified that participants in the intervention group who were male, aged 18 to 64 years, or living in urban areas were more likely to reduce their dose compared with those receiving usual care. Rates of substitution with other pain-relieving medications were not significantly different between groups. A post hoc reduction in all-cause mortality was observed in the intervention group.

There are explanatory differences between the current trial and previously successful deprescribing trials that used direct-to-patient education.^21,25^ First, pharmacists played a pivotal role in previous successful sedative-deprescribing trials (EMPOWER^21^ and D-PRESCRIBE^25^). In contrast, the TAPERING educational brochure was not delivered by a health care practitioner with whom the patient had rapport and trust.^29^ As trust is crucial in many patient-driven decisions, this may explain the failure of a government-led educational initiative.^30,31^ Second, factors associated with resistance to sedative deprescribing, including dependence and tolerance, may be more pronounced with opioids due to enhanced physical and psychosocial dependence,^32,33^ potentially explaining why the target reductions were not achieved in this trial. Third, EMPOWER^21^ and D-PRESCRIBE^25^ included tapering schedules that were faster than those used for opioids; thus, reductions in opioid doses should take longer to be observed.

There may be a number of reasons why the intervention failed to achieve the prespecified outcomes of prescription cessation, reduction to below 90 MME/d, or a 25% or greater dose reduction. First, the opioid crisis was well under way at the initiation of the study, meaning public awareness was high. Furthermore, the Manitoba government had already implemented several other strategies to curb opioid use, including restricting codeine sales and using pain specialists to support family physicians with opioid prescribing. Additionally, private insurers restricted high-dose opioid prescribing. Within this context, opioid use was already decreasing when this trial started, with Canada experiencing a 10% decrease in the quantity of opioids dispensed prior to trial onset in 2016 and 2017.^34^ The lack of effectiveness of this trial’s intervention on opioid cessation could be due to the study population being recalcitrant opioid consumers. Second, the brochure repeatedly advised people to talk to their health care practitioner about their opioid use and pain. However, at the time, health care practitioners had limited self-efficacy for deprescribing opioids.^35^ A new evidence-based opioid deprescribing guideline may help overcome this barrier.^36^ Third, subgroup analyses revealed that people were more likely to reduce their dose if they were male or aged 18 to 64 years, groups that visit their family practitioner less frequently.^37^ This aligns with previous studies in the primary care setting that found males were more likely to taper their opioids compared with females.^38^ Finally, people living in nonurban areas may have had difficulty accessing multidisciplinary pain clinics or the nonpharmacologic treatments mentioned in the brochure.^39^ While the results of this study are modest, they highlight how governments can successfully provide population-wide education on medication safety to reduce opioid demand. Future trials should investigate potential synergism between multicomponent policy interventions that target both patient and prescriber knowledge and behavior.

The TAPERING intervention produced a small yet significant reduction in all-cause mortality in the intervention group compared with the control group. One reason for this possible association relates to the brochure highlighting the increased risks from combining opioids with alcohol and/or sedative-hypnotics. This warning, coupled with the Manitoba government supplying thousands of free naloxone kits across the province, may have heightened awareness for securing and using a naloxone kit to treat accidental overdoses. Without linkage to detailed coroner reports, this explanation requires further investigation.

### Strengths and Limitations

Partnering government policy with academic evaluation was a strength of this trial; however, intrinsic limitations arose from the trial design. The collection of qualitative data to drive hypothesis generation ascertaining why the intervention worked in some groups and not others was not possible, nor were patient-reported outcomes, such as pain, function, quality of life, and adverse events, able to be measured. Some participants may not have received, opened, or correctly understood the mailed documentation. Randomization was clustered, and stratification at the clinic level was chosen to minimize potential bias, as all residents in the vicinity of a clinic were allocated to the same arm. Cross-contamination between groups through physicians with multiple practice locations or household contamination could have occurred. Administrative datasets are trustable sources for pharmacy dispensing claims; however, they cannot account for actual daily use of medication for individual patients or use of nonprescription opioids. There are inherent limitations to conducting a pragmatic trial. For this study, we were unable to determine whether participants in the intervention group did indeed receive, open, and understand the intervention information. While it was reassuring that only 40 brochures were returned as undeliverable, it is possible that intervention information was discarded rather than returned to the sender. Additionally, populations such as those experiencing homelessness or who were precariously housed may have been unable to access the intervention material, potentially limiting the effectiveness of the intervention across the whole province.

## Conclusions

In this cluster randomized clinical trial, direct-to-consumer educational material mailed to long-term opioid consumers did not lead to a significant difference in opioid cessation rate compared with usual care after 6 months. However, the intervention resulted in more adults reducing their opioid dose. The trial also showed that robust evaluation of opioid policy interventions is feasible using administrative data.

## Notes

Supplement 1.
Trial Protocol and Statistical Analysis Plan

Supplement 2. **eTable.** Morphine Milligram Equivalence Conversion Table

Supplement 3.
Data Sharing Statement

## References

[1] Canadian Institute for Health Information. Opioid-related harms in Canada. December 2018. Accessed May 14, 2019. https://www.cihi.ca/sites/default/files/document/opioid-related-harms-report-2018-en-web.pdf

[2] Gomes T, Tadrous M, Mamdani MM, Paterson JM, Juurlink DN. The burden of opioid-related mortality in the United States. JAMA Netw Open. 2018;1(2):e180217. doi:10.1001/jamanetworkopen.2018.0217 30646062 PMC6324425

[3] Special Advisory Committee on the Epidemic of Opioid Overdoses. Apparent opioid-related deaths in Canada (January 2016 to December 2018). 2019. Accessed July 19, 2019. https://www.canada.ca/en/public-health/services/publications/healthy-living/national-report-apparent-opioid-related-deaths-released-june-2018.html

[4] Canadian Centre on Substance Use and Addiction. Prescription Opioids (Canadian Drug Summary). 2020. Accessed July 19, 2019. https://www.ccsa.ca/prescription-opioids-canadian-drug-summary?_cldee=Y2FyYS50YW5uZW5iYXVtQHVtb250cmVhbC5jYQ%3d%3d&recipientid=contact-1b59d504131ee7118111480fcfeab9c1-d7e8afbc8c4544c3949cb9d70b275bc9&esid=985811e4-edc1-ea11-a812-000d3af42c56

[5] Health Canada. Government of Canada actions on opioids 2016 and 2017. 2017. Accessed September 1, 2019. https://www.canada.ca/content/dam/hc-sc/documents/services/publications/healthy-living/actions-opioids-2016-2017/Opioids-Response-Report-EN-FINAL.pdf

[6] Larochelle MR, Zhang F, Ross-Degnan D, Wharam JF. Rates of opioid dispensing and overdose after introduction of abuse-deterrent extended-release oxycodone and withdrawal of propoxyphene. JAMA Intern Med. 2015;175(6):978-987. doi:10.1001/jamainternmed.2015.0914 25895077

[7] Gomes T, Jain S, Paterson JM, Sketris I, Caetano P, Henry D; Canadian Network for Observational Drug Effect Studies (CNODES) Investigators. Trends and uptake of new formulations of controlled-release oxycodone in Canada. Pharmacoepidemiol Drug Saf. 2018;27(5):520-525. doi:10.1002/pds.4390 29359446 PMC5947657

[8] Patrick SW, Fry CE, Jones TF, Buntin MB. Implementation of prescription drug monitoring programs associated with reductions in opioid-related death rates. Health Aff (Millwood). 2016;35(7):1324-1332. doi:10.1377/hlthaff.2015.1496 27335101 PMC5155336

[9] Buchmueller TC, Carey C. The effect of prescription drug monitoring programs on opioid utilization in Medicare. Am Econ J Econ Policy. 2018;10(1):77-112. doi:10.1257/pol.20160094

[10] Meara E, Horwitz JR, Powell W, . State legal restrictions and prescription-opioid use among disabled adults. N Engl J Med. 2016;375(1):44-53. doi:10.1056/NEJMsa1514387 27332619 PMC4985562

[11] Chateau D, Enns M, Ekuma O, . Evaluation of the Manitoba IMP℞OVE Program. 2015. Accessed September 1, 2019. http://mchp-appserv.cpe.umanitoba.ca/reference//ImproveRx_report_website.pdf

[12] Sacarny A, Barnett ML, Le J, Tetkoski F, Yokum D, Agrawal S. Effect of peer comparison letters for high-volume primary care prescribers of quetiapine in older and disabled adults: a randomized clinical trial. JAMA Psychiatry. 2018;75(10):1003-1011. doi:10.1001/jamapsychiatry.2018.1867 30073273 PMC6233799

[13] Barnett ML, Gray J, Zink A, Jena AB. Coupling policymaking with evaluation—the case of the opioid crisis. N Engl J Med. 2017;377(24):2306-2309. doi:10.1056/NEJMp1710014 29236636

[14] Windmill J, Fisher E, Eccleston C, . Interventions for the reduction of prescribed opioid use in chronic non-cancer pain. Cochrane Database Syst Rev. 2013;(9):CD010323. doi:10.1002/14651858.CD010323 23996347

[15] Eccleston C, Fisher E, Thomas KH, . Interventions for the reduction of prescribed opioid use in chronic non-cancer pain. Cochrane Database Syst Rev. 2017;11(11):CD010323. 29130474 10.1002/14651858.CD010323.pub3 PMC6486018

[16] Volkow ND, Jones EB, Einstein EB, Wargo EM. Prevention and treatment of opioid misuse and addiction: a review. JAMA Psychiatry. 2019;76(2):208-216. doi:10.1001/jamapsychiatry.2018.3126 30516809

[17] Rubin R. Limits on opioid prescribing leave patients with chronic pain vulnerable. JAMA. 2019;321(21):2059-2062. doi:10.1001/jama.2019.5188 31034007

[18] Agnoli A, Xing G, Tancredi DJ, Magnan E, Jerant A, Fenton JJ. Association of dose tapering with overdose or mental health crisis among patients prescribed long-term opioids. JAMA. 2021;326(5):411-419. doi:10.1001/jama.2021.11013 34342618 PMC8335575

[19] Oliva EM, Bowe T, Manhapra A, . Associations between stopping prescriptions for opioids, length of opioid treatment, and overdose or suicide deaths in US veterans: observational evaluation. BMJ. 2020;368:m283. doi:10.1136/bmj.m283 32131996 PMC7249243

[20] Frank JW, Lovejoy TI, Becker WC, . Patient outcomes in dose reduction or discontinuation of long-term opioid therapy: a systematic review. Ann Intern Med. 2017;167(3):181-191. doi:10.7326/M17-0598 28715848

[21] Tannenbaum C, Martin P, Tamblyn R, Benedetti A, Ahmed S. Reduction of inappropriate benzodiazepine prescriptions among older adults through direct patient education: the EMPOWER cluster randomized trial. JAMA Intern Med. 2014;174(6):890-898. doi:10.1001/jamainternmed.2014.949 24733354

[22] Tannenbaum C, Farrell B, Shaw J, . An ecological approach to reducing potentially inappropriate medication use: Canadian deprescribing network. Can J Aging. 2017;36(1):97-107. doi:10.1017/S0714980816000702 28091333

[23] Turner JP, Caetano P, Tannenbaum C. Leveraging policy to reduce chronic opioid use by educating and empowering community dwelling adults: a study protocol for the TAPERING randomized controlled trial. Trials. 2019;20(1):412. doi:10.1186/s13063-019-3508-z 31288859 PMC6617933

[24] Schulz KF, Altman DG, Moher D; CONSORT Group. CONSORT 2010 Statement: updated guidelines for reporting parallel group randomised trials. BMC Med. 2010;8:18. doi:10.1186/1741-7015-8-18 20619135

[25] Martin P, Tamblyn R, Benedetti A, Ahmed S, Tannenbaum C. Effect of a pharmacist-led educational intervention on inappropriate medication prescriptions in older adults: the D-PRESCRIBE randomized clinical trial. JAMA. 2018;320(18):1889-1898. doi:10.1001/jama.2018.16131 30422193 PMC6248132

[26] Canadian Medication Appropriateness and Deprescribing Network. Opioid reduction. Updated 2023. Accessed September 1, 2019. https://www.deprescribingnetwork.ca/opioid-reduction

[27] Busse JW, Craigie S, Juurlink DN, . Guideline for opioid therapy and chronic noncancer pain. CMAJ. 2017;189(18):E659-E666. doi:10.1503/cmaj.170363 28483845 PMC5422149

[28] WHO Collaborating Centre for Drug Statistics Methodology. Guidelines for ATC Classification and DDD Assignment 2019. World Health Organisation; 2018.

[29] Zhang YZ, Turner JP, Martin P, Tannenbaum C. Does a consumer-targeted deprescribing intervention compromise patient-healthcare provider trust? Pharmacy (Basel). 2018;6(2):31. doi:10.3390/pharmacy6020031 29659488 PMC6024917

[30] Larson HJ, Heymann DL. Public health response to influenza A(H1N1) as an opportunity to build public trust. JAMA. 2010;303(3):271-272. doi:10.1001/jama.2009.2023 20085957

[31] Blair RA, Morse BS, Tsai LL. Public health and public trust: survey evidence from the Ebola virus disease epidemic in Liberia. Soc Sci Med. 2017;172:89-97. doi:10.1016/j.socscimed.2016.11.016 27914936

[32] Langford AV, Gnjidic D, Lin CC, . “The lesser of two evils”: a framework analysis of consumers’ perspectives on opioid deprescribing and the development of opioid deprescribing guidelines. Pain. 2021;162(11):2686-2692. doi:10.1097/j.pain.0000000000002270 33769364

[33] Kelley CJ, Niznik JD, Ferreri SP, . Patient perceptions of opioids and benzodiazepines and attitudes toward deprescribing. Drugs Aging. 2023;40(12):1113-1122. doi:10.1007/s40266-023-01071-z 37792262 PMC10768261

[34] Canadian Institute for Health Information. Pan-Canadian trends in the prescribing of opioids, 2012 to 2016. 2017. Accessed May 14, 2019. https://www.cihi.ca/sites/default/files/document/pan-canadian-trends-opioid-prescribing-2017-en-web.pdf

[35] Turner JP, Gagnon CL, Khuong NB, McDonald EG, Tannenbaum C. The impact of educational whiteboard videos on healthcare providers’ self-efficacy to deprescribe. J Am Geriatr Soc. 2023;71(5):E15-E18. doi:10.1111/jgs.18335 36971456

[36] Langford AV, Lin CC, Bero L, . Clinical practice guideline for deprescribing opioid analgesics: summary of recommendations. Med J Aust. 2023;219(2):80-89. doi:10.5694/mja2.52002 37356051

[37] Mukhtar TK, Bankhead C, Stevens S, . Factors associated with consultation rates in general practice in England, 2013-2014: a cross-sectional study. Br J Gen Pract. 2018;68(670):e370-e377. doi:10.3399/bjgp18X695981 29686130 PMC5916084

[38] Weimer MB, Hartung DM, Ahmed S, Nicolaidis C. A chronic opioid therapy dose reduction policy in primary care. Subst Abus. 2016;37(1):141-147. doi:10.1080/08897077.2015.1129526 26685018

[39] Peng P, Choiniere M, Dion D, ; STOPPAIN Investigators Group. Challenges in accessing multidisciplinary pain treatment facilities in Canada. Can J Anaesth. 2007;54(12):977-984. doi:10.1007/BF03016631 18056206

