# Supplementary material for: Government Direct-to-Consumer Education to Reduce Prescription Opioid Use: A Cluster Randomized Clinical Trial
Source: JAMA Netw Open. 2024 May 29;7(5):e2413698. doi: 10.1001/jamanetworkopen.2024.13698 (PMC11137632; doi:10.1001/jamanetworkopen.2024.13698)
Supplement: Supplement 1. — Trial Protocol and Statistical Analysis Plan [file jamanetwopen-e2413698-s001.pdf]

Supplementary File 1

Trial Protocol and Statistical Analysis Plan

This trial protocol has been provided by the authors to give readers additional information about their work. Protocol for: Turner JP, Halme AS, Caetano P, Tannenbaum C. Effectiveness of government-led direct-to-consumer education on reductions in chronic opioid use: The TAPERING cluster randomized controlled trial.

This supplement contains the following items:

1. Trial Protocols
    a. Original protocol -Page 2
    b. Final Protocol - Page 9
    c. Summary of Changes - Page 17
2. Statistical Analysis Plan - Page 19

Original Protocol

Using policy to reduce opioids by educating and empowering patients with chronic non-cancer pain: The TAPERING randomised trial.

(TAPERING: Trial Applying Policy to Eliminate or Reduce Inappropriate Narcotics in the General-population)

ABSTRACT:

Background: Opioids are often prescribed appropriately for acute pain, palliative care and cancer pain. However, the use of opioids to treat chronic non-cancer pain has grown steadily in the last two decades, despite limited evidence of benefit in this setting.[1] In parallel with the increased use, there has been an increase in opioid induced harm, with opioid related deaths tripling in Canada over the past decade.[2] This has caused the federal government to declare that Canada is in the middle of an opioid crisis.

In 2016, approximately 10% of the Canadian adult population consumed an opioid, costing the publicly funded drug plans over $200 Million. More than 13 Canadians being hospitalised each day due to opioid posioning.[2] Opioid related deaths have overtaken motor vehicle accidents fatalities, with over 2,500 Canadians dying as a direct result of opioids, compared to only 1,858 deaths from motor vehicle accidents.[3, 4] In the province of Manitoba, more than 10 people per 100,000 are hospitalised each day as a result of opioid toxicity.[2] Furthermore, approximately 10 people per 100,000 die each year as a result of consuming opioids.[2, 3]

When used to treat chronic non-cancer pain, opioids have been linked with increased risk of fractures, [5-7] myocardial infarction,[8, 9] sexual dysfunction in men,[10] and motor vehicle accidents.[11] In most instances, the association between opiate use and adverse effect increases with increasing dose and or duration of use.[1]

Deprescribing is the process of dose reduction and withdrawal of a medication, supervised by a health care professional, with the goal of improving patient outcomes.[12] The Canadian Guideline for Opioid Therapy and Chronic Non-cancer Pain was updated in 2017, and recommends that physicians trail tapering to the lowest possible dose, particularly or patients taking more than 90mg of oral-morphine-equivalents a day.[13] Despite this recommendation, the guideline acknowledges there is limited research demonstrating practical and effective interventions to reducing opioid use.

Researchers at Institute Universitaire de gériatrie de Montréal have demonstrated that patient empowerment can result in a significant reduction in use of inappropriate medications. In the EMPOWER trial, direct-to-consumer education about the potential harms of sedative-hypnotics led 62% of trial participants to discuss deprescribing with a healthcare provider, and 27% to discontinue chronic sedative-hypnotic use within 6 months.[14] This trial produced a significant reduction in inappropriate medication use, however, important questions remain unanswered: 1) Are the results externally reproducible on a large scale or were they affected by recruitment bias? 2) Is the intervention transferable between other classes of inappropriate medication, such as opioids for chronic non-cancer pain?

Hypothesis: It is hypothesised that posting a direct-to-consumer educational brochure to community dwelling adults will result in the reduction in opioid prescribing.

Primary aim:

1. To ***evaluate*** the effectiveness of a Manitoba Health policy in which direct-to-consumer educational brochures aimed at reducing inappropriate opioid use in chronic non-cancer pain are posted to 50% of the population, as measured by the deprescribing or dose reduction of opioids at three and six months compared to usual care.

METHODS:

Study Design

This is an analysis of a pragmatic policy intervention that will be rolled out by Manitoba Health in a staged approach to facilitate analysis of its effect. The policy will be implemented such that a randomized proportion of the population will be posted a direct-to-patient educational brochure first, with the remainder of the population receiving the intervention within the subsequent 12 months. This implementation was chosen to allow the intervention to be delivered and analysed at the patient level and compared to a control group receiving usual care. The randomized controlled trial design reduces potential for bias, while the pragmatic approach appreciates that not all patients will actively engage in the trial.

Ethics

The Manitoba government has authority to implement this trial as policy without requiring ethics approval. However, to conduct a scientific analysis of the policy implementation, scientific peer review and Research Ethics Board (REB) approval for the project is being sought through Institut universitaire de gériatrie de Montréal, Montréal, Quebec. This project is a province-wide scale up of a trial that has already been granted ethics approval from this REB. The previous trial has been conducted safely by this research group and, therefore, we consider this intervention to be low and negligible risk.
This trial will not involve direct contact between the research team and patients, pharmacists or physicians, therefore, individual consent will not be sought. No identifiable patient data will be released by Manitoba Health

Study population

The study population consists of the entire community dwelling population of Manitoba aged 18 years and over who are registered to receive pharmaceutical benefits with Manitoba Health. Using the Drug Program Information Network (DPIN), the Provincial Drugs Program Unit within Manitoba Health collects data of all medications dispensed in Manitoba (excluding hospital pharmacies, nursing stations ,ward stock and outpatient visits at CancerCare Manitoba). Using protocols previously developed by the Provincial Drugs Program Unit for the IMPRxOVE study, people who have had opioids dispensed can be identified.

Inclusion

Adults registered to receive pharmaceutical benefits in Manitoba, who are aged 18 years and over and who have received ≥90 days supply of opioids in the 6 months prior to the trial will be included. This will ensure chronic use and episodic use of opioids will be included.

Exclusion

People receiving palliative care, people with cancer, and people who receive opioids in hospital. As with the EMPOWER trial, people with dementia (identified by having received a prescription for memantine or a cholinersterase inhibitor in the previous 6 months) will be excluded.

Randomising and blinding

To reduce intervention bias, whereby there is contamination between participants in the intervention arm those in the control arm, this intervention will be delivered to geographical areas. Geographical areas will be identified using postal codes_and stratified to minimise contextual effects that may influence outcomes, such as age, sex, socio-economic status and population density.

A statistician, blinded to the intervention, will generate a random allocation sequence using computer-generated random digits. Geographical areas matched by postal code and population density will be randomised to either intervention arm or control at an allocation ratio of 1:1. People will be allocated to an arm in accordance with the geographical area of their residential address.

Blinding of the educational interventions is impossible for patients, pharmacists and family doctors. However, analysis will be carried out by a research staff member who is blinded to the allocation.

Intervention

All eligible patients living in the geographical area randomised to intervention will be posted the direct-to-consumer educational brochure and a cover letter. The cover letter will explain the context for the brochure, providing a public safety message with an overview of the benefits and harms of opioid use in Manitoba. The cover letter will ensure patients who are taking opioids appropriately, e.g. patients with cancer pain, understand the brochure is not directed at them.

Sample size

A sample size calculation is based on the number of patients required to identify a minimum 10% difference in cessation of opioids in the intervention arms compared to the control "usual care" arm (assuming there is a 5% discontinuation rate in the control arm, this will result in a minimum 15% absolute reduction in the prevalence of inappropriate medications). This is a conservative estimate, which is half the effect size observed in the EMPOWER trial (The EMPOWER trial achieved a 23% difference (27% reduction in the intervention arm, and 4.5% in the control arm)).[14] Based on a power of 80% and an α level of 0.05 (2 sided), we estimate that 343 participants will be needed for each arm. This sample size is achievable because there were over 14,000 adults in Manitoba who were dispensed opioids in 2016, excluding hospitalised and palliative care patients.

Data Collection

Data collection will occur through the Provincial Drugs Program Unit within Manitoba Health. Baseline data will be used to identify patients who meet inclusion criteria. Data will be extracted baseline and at three and six months following the educational intervention. All data will be anonymized before being provided to the research team.

Measurements and Definitions

Medication use will be measured using the DPIN pharmacy claims data for medications dispensed in Manitoba. Chronic opioid use will be defined as regular dispensing of ≥90 days of opioids within the previous 120 days. Episodic opioid use will be defined as ≥90 days of opioids in 121-180 days. Medications will be coded as International Non-proprietary Names and Anatomical Therapeutic Chemical (ATC) codes recommended by the World Health Organization,[15] and Drug Identification Numbers (DIN) according to Health Canada (available here https://health-products.canada.ca/dpd-bdpp/index-eng.jsp).[15]

Target Medications will be defined as:

- Opioids (N02A)

Alternatives to target medications will be defined as pharmacy claims for the following mediations that had not been claimed in the previous 12 months:

- other opioids (N02A), acetaminophen (N02BE), non-steroidal anti-inflammatory drugs (NSAIDs) (oral use (M01A) and topical use (M02AA)), and disease modifying antirehumatic drugs (DMARDS) (aminosalicylic acid and similar agents (A07EC), folic acid analogues (L01BA), selective immunosuppressants (L04AA), other immunosuppressants (L04AX), specific antirheumatic agents (M01C), aminoquinolines (P01BA)), gabapentin (N03AX12), pregabalin (N03AX16), low dose tricyclic antidepressants (N06AA), duloxetine (N06AX21) and carbamazepine (N303AF01)

The primary outcome, complete cessation of the opioid, will be assessed at the patient level using DPIN pharmacy claims data at three and six months following the intervention. An additional, 24 months of DPIN pharmacy claims data will be collected to determine any trends in prescribing before the intervention. Rates of discontinuation between the intervention arm and the control arm will be compared.

Statistical Analysis

Differences between baseline characteristics of intervention and control arms will be analysed using descriptive statistics, with Chi-square tests for categorical data and T-tests and Mann-Whitney-U test to compare the means of normally and non-normally distributed variables respectively.

The outcome of the primary aim, a reduction in opioid dispensing, will be analysed using an intention-to-treat approach. The unadjusted risk differences (with 95% confidence intervals) will be calculated via generalized estimating equations with a discontinuation as a binary outcome, assessed for each patient at three and six months following intervention and an identity link, using patients as the unit of analysis. The duration of the intervention effect will be analysed by comparing the reduction in prevalence of prescription refills on a month by month basis compared to baseline.

Payment

There will be no financial incentive to patients or family doctors to change prescribing. This prevents a potential ethical problem whereby appropriate medications may be deprescribed due to financial incentive. Altering prescribing remains the responsibility of the family physician based on clinical judgment and patient preferences.

Financial supplementation will be provided to Manitoba Health to cover the cost of some expenses involved with the project (e.g. printing and posting of patient brochures).

Outcome Measures

The primary outcome, complete cessation of target medication, will be assessed at the patient level using DPIN pharmacy claims data at three and six months following the intervention. Deprescribing will be defined as no opioid medication dispensed for a continuous 90 day period. Secondary outcomes include dose reduction and/or therapeutic switch. Dose reduction will be defined in two ways. Firstly, we will consider dose reduction as being a ≥25% reduction in the dose of opioids dispensed to participants when comparing the 6 months of the intervention, to the 6-months immediately prior to the intervention. Additionally, in accordance with the new Canadian Guideline for Opioid Therapy and Chronic Non-cancer Pain, we will investigate the proportion of patients who were receiving ≥90mg oral morphine equivalents at baseline who reduce their dose to <90mg oral morphine equivalents. New dispensing of a prescription for a lower strength, or supply of a reduced quantity of tablets/patches will be considered in the dose reduction calculation. Where substitution to an alternative opioid occurs, opioid doses will be converted to “oral morphine equivalents” to determine if a dose reduction occurred. A therapeutic switch will be indicated by a new dispensing for an alternative medication class as mentioned above.

OUTCOMES AND SIGNIFICANCE

Canada is in the middle of an opioid crisis. Opioids when abused, and even when used as directed can result in personal cost due to reduced quality of life, and health system costs through unnecessary medication expenses, and additional health system utilisation due to adverse drug events and hospitalisations.[16] This project is an important step in reducing inappropriate opioid use.

The proposed research will scale up a previously successful randomised controlled clinical trial. This will result in the reduction of inappropriate opioid use in Manitoba. Reducing inappropriate opioid use will improve health outcomes, whilst providing significant health system cost benefits. This research will build capacity in the area of primary care deprescribing, improving understanding about scaling up clinical trials to become provincial policy interventions.

TIMELINE

1. Identify geographical areas for stratification purposes
2. Randomize strata to either intervention arm or control
3. Identify patients in the intervention and control arms, and post the direct-to-consumer educational brochure to patients in the patient intervention arm
4. Collect three and six month outcome data, along with the data from 24 months prior to intervention to determine baseline trends

SUMMARY OF OUTCOME DATA TO BE COLLECTED FROM THE DPIN

**DPIN Pharmacy Claims: Patient Level Data.**	
**Data to collect**	**Outcome Measure**
For each target medication<br>- Dispense dates<br>- Strength & dose<br>- Quantity<br>- Refills available<br>- Physician ID	For each target medication<br>- Change in dose<br>- Cessation<br>- Reduced frequency of prescription refill<br>- Physician ID (To determine the specialty of the physician)
Alternative medications<br>- Dispense dates<br>- Strength & dose<br>- Quantity<br>- Refills available<br>- Physician ID	For each alternative medication<br>- Dose<br>- Duration<br>- Dispense date in relation to target medication<br>- Number of dispensings<br>- Usage – PRN or regular

REFERENCES:

1. Chou R, Turner JA, Devine EB, et al. The effectiveness and risks of long-term opioid therapy for chronic pain: a systematic review for a National Institutes of Health Pathways to Prevention Workshop. *Ann Intern Med* 2015;162:276-86.
2. Canadian Institute for Health Information Canadian Centre on Substance Abuse. *Hospitalizations and Emergency Department Visits Due to Opioid Poisoning in Canada.* Ottawa, ON: CIHI; 2016.
3. Health Canada. *National report: apparent opioid-related deaths (2016)*: Health Canada 2017.
4. Transort Canada. Canadian Motor Vehicle Traffic Collision Statistics 2015. 2017.
5. Saunders KW, Dunn KM, Merrill JO, et al. Relationship of opioid use and dosage levels to fractures in older chronic pain patients. *J Gen Intern Med* 2010;25:310-5.
6. Miller M, Sturmer T, Azrael D, Levin R, Solomon DH. Opioid analgesics and the risk of fractures in older adults with arthritis. *J Am Geriatr Soc* 2011;59:430-8.
7. Li L, Setoguchi S, Cabral H, Jick S. Opioid use for noncancer pain and risk of fracture in adults: a nested case-control study using the general practice research database. *Am J Epidemiol* 2013;178:559-69.
8. Carman WJ, Su S, Cook SF, Wurzelmann JI, McAfee A. Coronary heart disease outcomes among chronic opioid and cyclooxygenase-2 users compared with a general population cohort. *Pharmacoepidemiol Drug Saf* 2011;20:754-62.
9. Li L, Setoguchi S, Cabral H, Jick S. Opioid use for noncancer pain and risk of myocardial infarction amongst adults. *J Intern Med* 2013;273:511-26.
10. Deyo RA, Smith DH, Johnson ES, et al. Prescription opioids for back pain and use of medications for erectile dysfunction. *Spine (Phila Pa 1976)* 2013;38:909-15.
11. Gomes T, Redelmeier DA, Juurlink DN, Dhalla IA, Camacho X, Mamdani MM. Opioid dose and risk of road trauma in Canada: a population-based study. *JAMA Intern Med* 2013;173:196-201.
12. Reeve E, Gnjidic D, Long J, Hilmer S. A systematic review of the emerging definition of 'deprescribing' with network analysis: implications for future research and clinical practice. *Br J Clin Pharmacol* 2015;80:1254-68.
13. Busse JW, Craigie S, Juurlink DN, et al. Guideline for opioid therapy and chronic noncancer pain. *Can Med Assoc J* 2017;189:E659-E66.
14. Tannenbaum C, Martin P, Tamblyn R, Benedetti A, Ahmed S. Reduction of inappropriate benzodiazepine prescriptions among older adults through direct patient education: the EMPOWER cluster randomized trial. *JAMA Intern Med* 2014;174:890-8.
15. WHO Collaborating Centre for Drug Statistics Methodology. *Guidelines for ATC classification and DDD assignment, 2012.* Oslo2011.
16. Morgan SG, Hunt J, Rioux J, Proulx J, Weymann D, Tannenbaum C. Frequency and cost of potentially inappropriate prescribing for older adults: a cross-sectional study. *CMAJ Open* 2016;4:E346-E51.

Final Protocol

<u>Using policy to reduce opioids by educating and empowering patients with chronic non-cancer pain: The TAPERING randomised trial.</u>

(TAPERING: Trial Applying Policy to Eliminate or Reduce Inappropriate Narcotics in the General-population)

Revision No.1

ABSTRACT:

<u>Background:</u> Opioids are often prescribed appropriately for acute pain, palliative care and cancer pain. However, the use of opioids to treat chronic non-cancer pain has grown steadily in the last two decades, despite limited evidence of benefit in this setting.[1] In parallel with the increased use, there has been an increase in opioid induced harm, with opioid related deaths tripling in Canada over the past decade.[2] This has caused the federal government to declare that Canada is in the middle of an opioid crisis.

In 2016, approximately 10% of the Canadian adult population consumed an opioid, costing the publicly funded drug plans over $200 Million. More than 13 Canadians being hospitalised each day due to opioid posioning.[2] Opioid related deaths have overtaken motor vehicle accidents fatalities, with over 2,500 Canadians dying as a direct result of opioids, compared to only 1,858 deaths from motor vehicle accidents.[3, 4] In the province of Manitoba, more than 10 people per 100,000 are hospitalised each day as a result of opioid toxicity.[2] Furthermore, approximately 10 people per 100,000 die each year as a result of consuming opioids.[2, 3]

When used to treat chronic non-cancer pain, opioids have been linked with increased risk of fractures, [5-7] myocardial infarction,[8, 9] sexual dysfunction in men,[10] and motor vehicle accidents.[11] In most instances, the association between opiate use and adverse effect increases with increasing dose and or duration of use.[1]

Deprescribing is the process of dose reduction and withdrawal of a medication, supervised by a health care professional, with the goal of improving patient outcomes.[12] The Canadian Guideline for Opioid Therapy and Chronic Non-cancer Pain was updated in 2017, and recommends that physicians trail tapering to the lowest possible dose, particularly or patients taking more than 90mg of oral-morphine-equivalents a day.[13] Despite this recommendation, the guideline acknowledges there is limited research demonstrating practical and effective interventions to reducing opioid use.

Researchers at Institute Universitaire de gériatrie de Montréal have demonstrated that patient empowerment can result in a significant reduction in use of inappropriate medications. In the EMPOWER trial, direct-to-consumer education about the potential harms of sedative-hypnotics led 62% of trial participants to discuss deprescribing with a healthcare provider, and 27% to discontinue chronic sedative-hypnotic use within 6 months.[14] This trial produced a significant reduction in inappropriate medication use, however, important questions remain unanswered: 1) Are the results externally reproducible on a large scale or were they affected by recruitment bias? 2) Is the intervention transferable between other classes of inappropriate medication, such as opioids for chronic non-cancer pain?

<u>Hypothesis:</u> It is hypothesised that posting a direct-to-consumer educational brochure to community dwelling adults will result in the reduction in opioid prescribing.

Primary aim:

2. To ***evaluate*** the effectiveness of a Manitoba Health policy in which direct-to-consumer educational brochures aimed at reducing inappropriate opioid use in chronic non-cancer pain are posted to 50% of the population, as measured by the deprescribing or dose reduction of opioids at three and six months compared to usual care.

METHODS:

Study Design

This is an analysis of a pragmatic policy intervention that will be rolled out by Manitoba Health in a staged approach to facilitate analysis of its effect. This is a pragmatic, prospective, cluster randomised, parallel-arm controlled trial, comparing receipt of a direct-to-patient educational brochure in the mail (intervention arm) to usual care (control arm). A cluster design was chosen to prevent contamination between the intervention and control arms amongst patients who attended the same family medicine clinics (each family medicine clinic forms a cluster unit). By randomizing individuals according to practice unit, the cluster design reduces the potential for bias if a physician has patients from both the intervention and control arms, as the intervention group receives a letter and direct-to-patient educational brochure with instructions to talk to their family physician to reduce their opioid use. The policy will be implemented such that a randomized proportion of the population will be posted a direct-to-patient educational brochure first, with the remainder of the population receiving the intervention within the subsequent 12 months. This implementation was chosen to allow the intervention to be delivered and analysed at the patient level and compared to a control group receiving usual care. The randomized controlled trial design reduces potential for bias, while the pragmatic approach appreciates that not all patients will actively engage in the trial.

Ethics

The Manitoba government has authority to implement this trial as policy without requiring ethics approval. This project is a province-wide scale up of a trial that has already been granted ethics approval from this REB. The previous trial has been conducted safely by this research group and, therefore, we consider this intervention to be low and negligible risk.
This trial will not involve direct contact between the research team and patients, pharmacists or physicians, therefore, individual consent will not be sought. No identifiable patient data will be released by Manitoba Health
The trial protocol was approved by the Research Ethics Board of the Centre de Recherche de l'Institut Universitaire de gériatrie de Montréal, Canada, as part of the Centre intégré universitaire en santé et services sociaux du Centre-Sud de l'île de Montréal, Canada on the 20th of July 2017 (CER VN 17-18-25) (ClinicalTrials.gov identifier: NCT03400384). Consent to conduct this policy evaluation was provided by the Executive Director of the Provincial Drug Programs Branch, the Health Information Privacy Committee of Manitoba Health, Seniors and Active Living (MHSAL), and the Manitoba Monitored Drugs Review Committee.

Study population

The study population consists of the entire community dwelling population of Manitoba aged 18 years and over who are registered to receive pharmaceutical benefits with Manitoba Health. Using the Drug Program Information Network (DPIN), the Provincial Drugs Program Unit within Manitoba Health collects data of all medications dispensed in Manitoba (excluding

hospital pharmacies, nursing stations ,ward stock and outpatient visits at CancerCare Manitoba). Using protocols previously developed by the Provincial Drugs Program Unit for the IMPRxOVE study, people who have had opioids dispensed can be identified.

Inclusion

Adults registered to receive pharmaceutical benefits in Manitoba, who are aged 18 years and over and who have received ≥90 days supply of opioids in the 120 days prior to the trial will be included. This will ensure chronic use of opioids will be included. The supply of opioids will be calculated as the sum of all prescriptions dispensed within Manitoba for the following opioids: fentanyl, hydromorphone, meperidine (pethidine), morphine and oxycodone. Codeine and Tramadol will be excluded from this calculation.

Exclusion

People receiving palliative care, people with cancer, and people who receive opioids in hospital. As with the EMPOWER trial, people with dementia (identified by having received a prescription for memantine or a cholinersterase inhibitor in the previous 6 months) will be excluded. Additionally, people living in nursing homes will be excluded.

Randomising and blinding

To reduce intervention bias, whereby there is contamination between participants in the intervention arm those in the control arm, this intervention will be delivered in clusters of family medication clinics identified using postal codes from each physician's registered practicing address. Participants will be allocated to their primary physician, and the family medicine clinics will be stratified to minimise contextual effects that may influence outcomes, such as age, sex, socio-economic status and population density. Six cluster strata of prescribing physicians will be created using a computer algorithm: five strata according to the number of physicians registered at an individual six-digit postal code (1-10, 11-20, 21-30, 31-40, >40), and a sixth stratum for prescribers with no identifiable practicing address (figure 1). A statistician, blinded to the intervention, will generate a random allocation sequence using computer-generated random digits. Family medicine clinics will be randomised to either intervention arm or control at an allocation ratio of 1:1. People will be allocated to an arm in accordance with their family medicine clinic.
Blinding of the educational interventions is impossible for patients, pharmacists and family doctors. However, analysis will be carried out by a research staff member who is blinded to the allocation.

Intervention

All eligible patients randomised to intervention will be posted the direct-to-consumer educational brochure and a cover letter. The cover letter will explain the context for the brochure, providing a public safety message with an overview of the benefits and harms of opioid use in Manitoba. The cover letter will ensure patients who are taking opioids appropriately, e.g. patients with cancer pain, understand the brochure is not directed at them.

Sample size

A sample size calculation is based on the number of patients required to identify a minimum 10% difference in cessation of opioids in the intervention arms compared to the control "usual

care" arm (assuming there is a 5% discontinuation rate in the control arm, this will result in a minimum 15% absolute reduction in the prevalence of inappropriate medications). This is a conservative estimate, which is half the effect size observed in the EMPOWER trial (The EMPOWER trial achieved a 23% difference (27% reduction in the intervention arm, and 4.5% in the control arm)).[14] Based on a power of 80% and an α level of 0.05 (2 sided), we estimate that 343 participants will be needed for each arm. This sample size is achievable because there were over 14,000 adults in Manitoba who were dispensed opioids in 2016, excluding hospitalised and palliative care patients.

Data Collection

Data collection will occur through the Provincial Drugs Program Unit within Manitoba Health. Baseline data will be used to identify patients who meet inclusion criteria. Data will be extracted baseline and six months following the educational intervention. All data will be anonymized before being provided to the research team.

Measurements and Definitions

Medication use will be measured using the DPIN pharmacy claims data for medications dispensed in Manitoba. Chronic opioid use will be defined as regular dispensing of ≥90 days of opioids within the previous 120 days. Medications will be coded as International Non-proprietary Names and Anatomical Therapeutic Chemical (ATC) codes recommended by the World Health Organization,[15] and Drug Identification Numbers (DIN) according to Health Canada (available here https://health-products.canada.ca/dpd-bdpp/index-eng.jsp).[15]
Target Medications will be defined as:

- Opioids (N02A)

Alternatives to target medications will be defined as pharmacy claims for the following mediations that had not been claimed in the previous 12 months:

- other opioids (N02A), acetaminophen (N02BE), non-steroidal anti-inflammatory drugs (NSAIDs) (oral use (M01A) and topical use (M02AA)), and disease modifying antirehumatic drugs (DMARDS) (aminosalicylic acid and similar agents (A07EC), folic acid analogues (L01BA), selective immunosuppressants (L04AA), other immunosuppressants (L04AX), specific antirheumatic agents (M01C), aminoquinolines (P01BA)), gabapentin (N03AX12), pregabalin (N03AX16), low dose tricyclic antidepressants (N06AA), duloxetine (N06AX21) and carbamazepine (N303AF01)

The primary outcome, complete cessation of the opioid, will be assessed at the patient level using DPIN pharmacy claims data at three and six months following the intervention. An additional, 24 months of DPIN pharmacy claims data will be collected to determine any trends in prescribing before the intervention. Rates of discontinuation between the intervention arm and the control arm will be compared.

Statistical Analysis

Differences between baseline characteristics of intervention and control arms will be analysed using descriptive statistics, with Chi-square tests for categorical data and T-tests and Mann-Whitney-U test to compare the means of normally and non-normally distributed variables respectively.

The outcome of the primary aim, a reduction in opioid dispensing, will be analysed using an intention-to-treat approach. The unadjusted risk differences (with 95% confidence intervals) will be calculated via generalized estimating equations with a discontinuation as a binary outcome, assessed for each patient at six months following intervention and an identity link, using patients as the unit of analysis. The same method will be applied for secondary outcomes. A p value of <0.05 will be considered as statistically significant.

Payment

There will be no financial incentive to patients or family doctors to change prescribing. This prevents a potential ethical problem whereby appropriate medications may be deprescribed due to financial incentive. Altering prescribing remains the responsibility of the family physician based on clinical judgment and patient preferences.

Financial supplementation will be provided to Manitoba Health to cover the cost of some expenses involved with the project (e.g. printing and posting of patient brochures).

Outcome Measures

The primary outcome, complete cessation of target medication, will be assessed at the patient level using DPIN pharmacy claims data at three and six months following the intervention. Deprescribing will be defined as no opioid medication dispensed for a continuous 60 day period.

Secondary outcomes include dose reduction and/or therapeutic switch. Dose reduction will be defined in two ways. Firstly, we will consider dose reduction as being a ≥25% reduction in the dose of opioids dispensed to participants when comparing the 6 months of the intervention, to the 6-months immediately prior to the intervention. Additionally, in accordance with the new Canadian Guideline for Opioid Therapy and Chronic Non-cancer Pain, we will investigate the proportion of patients who were receiving ≥90mg oral morphine equivalents at baseline who reduce their dose to <90mg oral morphine equivalents. New dispensing of a prescription for a lower strength, or supply of a reduced quantity of tablets/patches will be considered in the dose reduction calculation. Where substitution to an alternative opioid occurs, opioid doses will be converted to "oral morphine equivalents" to determine if a dose reduction occurred. A therapeutic switch will be indicated by a new dispensing for an alternative medication class as mentioned above.

OUTCOMES AND SIGNIFICANCE

Canada is in the middle of an opioid crisis. Opioids when abused, and even when used as directed can result in personal cost due to reduced quality of life, and health system costs through unnecessary medication expenses, and additional health system utilisation due to adverse drug events and hospitalisations.[16] This project is an important step in reducing inappropriate opioid use.

The proposed research will scale up a previously successful randomised controlled clinical trial. This will result in the reduction of inappropriate opioid use in Manitoba. Reducing inappropriate opioid use will improve health outcomes, whilst providing significant health system cost benefits. This research will build capacity in the area of primary care deprescribing, improving understanding about scaling up clinical trials to become provincial policy interventions.

TIMELINE

5. Identify geographical areas for stratification purposes
6. Randomize strata to either intervention arm or control
7. Identify patients in the intervention and control arms, and post the direct-to-consumer educational brochure to patients in the patient intervention arm
8. Collect three and six month outcome data, along with the data from 24 months prior to intervention to determine baseline trends

SUMMARY OF OUTCOME DATA TO BE COLLECTED FROM THE DPIN

**DPIN Pharmacy Claims: Patient Level Data.**	
**Data to collect**	**Outcome Measure**
For each target medication<br>- Dispense dates<br>- Strength & dose<br>- Quantity<br>- Refills available<br>- Physician ID	For each target medication<br>- Change in dose<br>- Cessation<br>- Reduced frequency of prescription refill<br>- Physician ID (To determine the specialty of the physician)
Alternative medications<br>- Dispense dates<br>- Strength & dose<br>- Quantity<br>- Refills available<br>- Physician ID	For each alternative medication<br>- Dose<br>- Duration<br>- Dispense date in relation to target medication<br>- Number of dispensings<br>- Usage – PRN or regular

Figure 1. A flow chart for the study process.

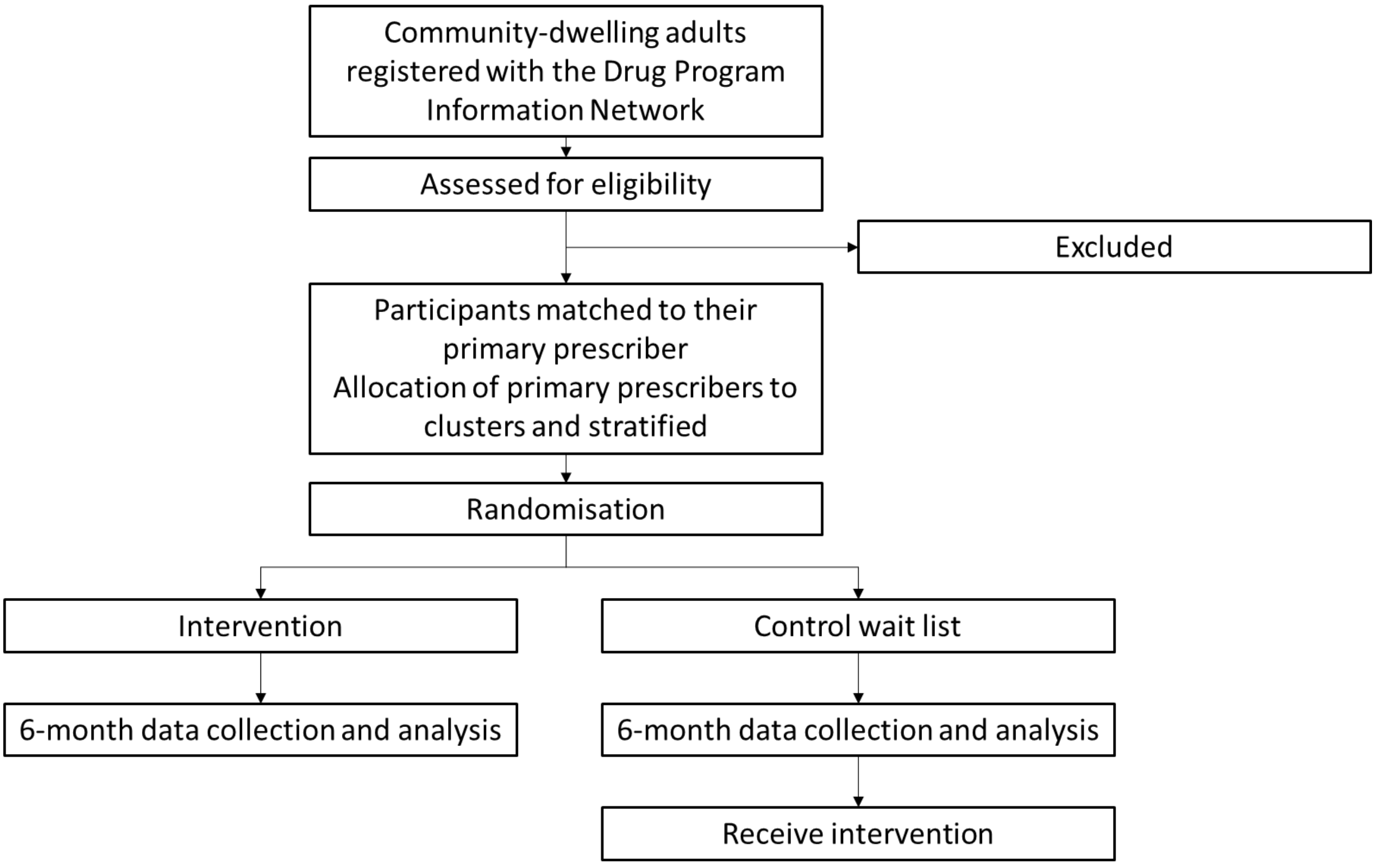

REFERENCES:

1. Chou R, Turner JA, Devine EB, et al. The effectiveness and risks of long-term opioid therapy for chronic pain: a systematic review for a National Institutes of Health Pathways to Prevention Workshop. *Ann Intern Med*. Feb 17 2015;162(4):276-86. doi:10.7326/M14-2559
2. Canadian Institute for Health Information Canadian Centre on Substance Abuse. *Hospitalizations and Emergency Department Visits Due to Opioid Poisoning in Canada*. 2016. https://secure.cihi.ca/free_products/Opioid%20Poisoning%20Report%20%20EN.pdf
3. Health Canada. *National report: apparent opioid-related deaths (2016)*. 2017. Accessed 05/07/2017. https://www.canada.ca/en/health-canada/services/substance-abuse/prescription-drug-abuse/opioids/national-report-apparent-opioid-related-deaths.html#def
4. Transort Canada. Canadian Motor Vehicle Traffic Collision Statistics 2015. 2017;
5. Saunders KW, Dunn KM, Merrill JO, et al. Relationship of opioid use and dosage levels to fractures in older chronic pain patients. journal article. *J Gen Intern Med*. Apr 2010;25(4):310-5. doi:10.1007/s11606-009-1218-z
6. Miller M, Sturmer T, Azrael D, Levin R, Solomon DH. Opioid analgesics and the risk of fractures in older adults with arthritis. *J Am Geriatr Soc*. Mar 2011;59(3):430-8. doi:10.1111/j.1532-5415.2011.03318.x
7. Li L, Setoguchi S, Cabral H, Jick S. Opioid use for noncancer pain and risk of fracture in adults: a nested case-control study using the general practice research database. *Am J Epidemiol*. Aug 15 2013;178(4):559-69. doi:10.1093/aje/kwt013
8. Carman WJ, Su S, Cook SF, Wurzelmann JI, McAfee A. Coronary heart disease outcomes among chronic opioid and cyclooxygenase-2 users compared with a general population cohort. *Pharmacoepidemiol Drug Saf*. Jul 2011;20(7):754-62. doi:10.1002/pds.2131
9. Li L, Setoguchi S, Cabral H, Jick S. Opioid use for noncancer pain and risk of myocardial infarction amongst adults. *J Intern Med*. May 2013;273(5):511-26. doi:10.1111/joim.12035
10. Deyo RA, Smith DH, Johnson ES, et al. Prescription opioids for back pain and use of medications for erectile dysfunction. *Spine (Phila Pa 1976)*. May 15 2013;38(11):909-15. doi:10.1097/BRS.0b013e3182830482
11. Gomes T, Redelmeier DA, Juurlink DN, Dhalla IA, Camacho X, Mamdani MM. Opioid dose and risk of road trauma in Canada: a population-based study. *JAMA Intern Med*. Feb 11 2013;173(3):196-201. doi:10.1001/2013.jamainternmed.733
12. Reeve E, Gnjidic D, Long J, Hilmer S. A systematic review of the emerging definition of 'deprescribing' with network analysis: implications for future research and clinical practice. *Br J Clin Pharmacol*. 2015;80(6):1254-1268. doi:10.1111/bcp.12732
13. Busse JW, Craigie S, Juurlink DN, et al. Guideline for opioid therapy and chronic noncancer pain. *Can Med Assoc J*. 2017;189(18):E659-E666. doi:10.1503/cmaj.170363
14. Tannenbaum C, Martin P, Tamblyn R, Benedetti A, Ahmed S. Reduction of inappropriate benzodiazepine prescriptions among older adults through direct patient education: the EMPOWER cluster randomized trial. *JAMA Intern Med*. Jun 2014;174(6):890-8. doi:10.1001/jamainternmed.2014.949
15. WHO Collaborating Centre for Drug Statistics Methodology. *Guidelines for ATC classification and DDD assignment, 2012.* 2011.
16. Morgan SG, Hunt J, Rioux J, Proulx J, Weymann D, Tannenbaum C. Frequency and cost of potentially inappropriate prescribing for older adults: a cross-sectional study. *CMAJ Open*. June 22, 2016 2016;4(2):E346-E351. doi:10.9778/cmajo.20150131

Summary of protocol changes

Approval for the TAPERING trial was sought and approved by the Research Ethics Board of the Centre de Recherche de l'Institut Universitaire de gériatrie de Montréal, Canada, as part of the Centre intégré universitaire en santé et services sociaux du Centre-Sud de l'île de Montréal, Canada, on the 20th of July 2017 (CER VN 17-18-25) (ClinicalTrials.gov identifier: NCT03400384).

This was the first step in a series of approvals. The protocol was also reviewed and ultimately approved by the Executive Director of the Provincial Drug Programs Branch, the Health Information Privacy Committee of Manitoba Health, Seniors and Active Living (MHSAL), and the Manitoba Monitored Drugs Review Committee.

During these approval processes, a number of changes were made to improve the trial and align it with the different contexts at play within Manitoba (e.g. data availability, staffing resources). Below is a summary of the changes and the rationale for the changes.

1. Cluster randomisation: The trial was changed from randomisation at the patient level, to cluster randomisation at the level of the family medicine clinic of the patient's primary physician. This change was made to reduce the potential for bias that may occur if a physician or another colleague within the same family clinic had some patients enrolled into the intervention arm and others in the usual care wait list arm. It was considered that the lessons learned from the intervention arm could be applied to the usual care patients. Randomisation was adjusted to account for clusters as opposed to individuals.

2. The original protocol proposed mailing the direct-to-patient educational brochure to people who were chronic users of opioids (defined as ≥90 days dispensed within the previous 120 days) as well as episodic users (defined as ≥90 days dispensed within the previous six months). There was concern that episodic users who might have already stopped their opioid would receive the intervention and become confused by the timing of the message. Thus, it was decided to focus only on recent chronic use and not to mail out brochures to people with episodic use.

3. The Manitoba Monitored Drug Review Committee recommended against mailing brochures to patients who were prescribed codeine or tramadol because ongoing work was already focusing on the prescribing of these medications. Consequently, both codeine and tramadol were excluded from the calculation to determine inclusion.

4. The Drug Program Information Network at the Provincial Drugs Program Unit within Manitoba Health has the ability to identify patients who are residents from long term

care. To align with the original EMPOWER trial, the decision was made to exclude these people.

5. The p value for significance was defined: A p value of <0.05 will be considered as statistically significant.

6. Originally a three and six month analysis was planned, however, due to staffing considerations within the department the interim three month analysis was removed.

7. The definition for deprescribing was revised from 90 days without an opioid prescription being dispensed to 60 days to reflect that opioids are dispensed as a maximum of 30 days at a time.

Statistical Analysis Plan as per Final Protocol

Data Collection

Data collection will occur through the Provincial Drugs Program Unit within Manitoba Health. Baseline data will be used to identify patients who meet inclusion criteria. Data will be extracted baseline and six months following the educational intervention. All data will be anonymized before being provided to the research team.

Measurements and Definitions

Medication use will be measured using the DPIN pharmacy claims data for medications dispensed in Manitoba. Chronic opioid use will be defined as regular dispensing of ≥90 days of opioids within the previous 120 days. Medications will be coded as International Non-proprietary Names and Anatomical Therapeutic Chemical (ATC) codes recommended by the World Health Organization,[15] and Drug Identification Numbers (DIN) according to Health Canada (available here https://health-products.canada.ca/dpd-bdpp/index-eng.jsp).[15]
Target Medications will be defined as:

- Opioids (N02A)

Alternatives to target medications will be defined as pharmacy claims for the following mediations that had not been claimed in the previous 12 months:

- other opioids (N02A), acetaminophen (N02BE), non-steroidal anti-inflammatory drugs (NSAIDs) (oral use (M01A) and topical use (M02AA)), and disease modifying antirehumatic drugs (DMARDS) (aminosalicylic acid and similar agents (A07EC), folic acid analogues (L01BA), selective immunosuppressants (L04AA), other immunosuppressants (L04AX), specific antirheumatic agents (M01C), aminoquinolines (P01BA)), gabapentin (N03AX12), pregabalin (N03AX16), low dose tricyclic antidepressants (N06AA), duloxetine (N06AX21) and carbamazepine (N303AF01)

The primary outcome, complete cessation of the opioid, will be assessed at the patient level using DPIN pharmacy claims data at three and six months following the intervention. An additional, 24 months of DPIN pharmacy claims data will be collected to determine any trends in prescribing before the intervention. Rates of discontinuation between the intervention arm and the control arm will be compared.

Statistical Analysis

Differences between baseline characteristics of intervention and control arms will be analysed using descriptive statistics, with Chi-square tests for categorical data and T-tests and Mann-Whitney-U test to compare the means of normally and non-normally distributed variables respectively.
The outcome of the primary aim, a reduction in opioid dispensing, will be analysed using an intention-to-treat approach. The unadjusted risk differences (with 95% confidence intervals) will be calculated via generalized estimating equations with a discontinuation as a binary outcome, assessed for each patient at six months following intervention and an identity link, using patients as the unit of analysis. The same method will be applied for secondary outcomes. A p value of <0.05 will be considered as statistically significant.

Outcome Measures

The primary outcome, complete cessation of target medication, will be assessed at the patient level using DPIN pharmacy claims data at three and six months following the intervention. Deprescribing will be defined as no opioid medication dispensed for a continuous 60 day period.

Secondary outcomes include dose reduction and/or therapeutic switch. Dose reduction will be defined in two ways. Firstly, we will consider dose reduction as being a ≥25% reduction in the dose of opioids dispensed to participants when comparing the 6 months of the intervention, to the 6-months immediately prior to the intervention. Additionally, in accordance with the new Canadian Guideline for Opioid Therapy and Chronic Non-cancer Pain, we will investigate the proportion of patients who were receiving ≥90mg oral morphine equivalents at baseline who reduce their dose to <90mg oral morphine equivalents. New dispensing of a prescription for a lower strength, or supply of a reduced quantity of tablets/patches will be considered in the dose reduction calculation. Where substitution to an alternative opioid occurs, opioid doses will be converted to “oral morphine equivalents” to determine if a dose reduction occurred. A therapeutic switch will be indicated by a new dispensing for an alternative medication class as mentioned above.
