# Supplementary material for: Government Direct-to-Consumer Education to Reduce Prescription Opioid Use: A Cluster Randomized Clinical Trial
Source: JAMA Netw Open. 2024 May 29;7(5):e2413698. doi: 10.1001/jamanetworkopen.2024.13698 (PMC11137632; doi:10.1001/jamanetworkopen.2024.13698)
Supplement: Supplement 2. — eTable. Morphine Milligram Equivalence Conversion Table [file jamanetwopen-e2413698-s002.pdf]

Supplementary Online Content

Turner JP, Halme AS, Caetano P, Langford A, Tannenbaum C. Government direct-to-consumer education to reduce prescription opioid use: a cluster randomized clinical trial. *JAMA Netw Open*. 2024;7(5):e2413698. doi:10.1001/jamanetworkopen.2024.13698

eTable. Morphine Milligram Equivalence Conversion Table

This supplementary material has been provided by the authors to give readers additional information about their work.

© 2024 Turner JP et al. *JAMA Network Open*.

eTable. Morphine Milligram Equivalence (MME) Conversion Table

This table is based on oral dosing for chronic non-cancer pain as per the Canadian Guideline for Safe and Effective Use of Opioids or Chronic Non-Cancer Pain.

Opioid	Convert to oral morphine milligram equivalent by multiplying by
Hydromorphone	5.0
Oxycodone	1.5
Meperidine	0.1
Transcutaneous fentanyl	100 (i.e. 25mcg/hour → 600mcg/day → 60mg morphine /day)

Reference: Busse JW, Craigie S, Juurlink DN, et al. Guideline for opioid therapy and chronic noncancer pain. *Cmaj*. May 8 2017;189(18):E659-e666. doi:10.1503/cmaj.170363

© 2024 Turner JP et al. *JAMA Network Open*.
