# Supplementary material for: Government Direct-to-Consumer Education to Reduce Prescription Opioid Use: A Cluster Randomized Clinical Trial
Source: JAMA Netw Open. 2024 May 29;7(5):e2413698. doi: 10.1001/jamanetworkopen.2024.13698 (PMC11137632; doi:10.1001/jamanetworkopen.2024.13698)
Supplement: Supplement 3. — Data Sharing Statement [file jamanetwopen-e2413698-s003.pdf]

Data Sharing Statement

Turner. Government Direct-to-Consumer Education to Reduce Prescription Opioid Use. *JAMA Netw Open*. Published May 29, 2024. doi:10.1001/jamanetworkopen.2024.13698

Data
Data available: No

Additional Information
Explanation for why data not available: Individual participant data was collected and anonymized by the Provincial Drug Programs Branch within the Manitoba Health, Seniors and Active Living Department of the Government of Manitoba and is therefore not available for sharing.
